# Hospitals

**Published:** 1874-10

**Authors:** 


### Hospitals.

***The Central Free Dispensary.*** Department of Gynæcology and Obstetrics. Service of Dr. P. ADOLPHUS.

DYSMENORRHŒA.—Previous to menstruation, where there has been dysmenorrhœa, the pain in the back and hypogastrium is greatly relieved by the use of a spear-pointed scarificator (Buttles'), plunging it into the cervix, and giving it a rapid turn before removal, which causes a very free flow of blood, should congestion exist. If leeches are employed, they should be applied to the perineum, or the inner aspect of the thighs, and not to the vagina or os uteri, for in certain patients the blood will sometimes continue oozing out for several days, causing trouble and annoyance both to physician and patient.

Should the hypogastric pain be severe, blistering tissue, or several applications of the compound tincture of iodine, over that region, will afford relief. In some cases, after the menstrual period has passed, the cervix may be dilated by Nott's Dilator, or by bougies; occasionally, if mechanical obstruction exists, if the os be very small or conical, it is well to employ Emmet's method of incision of the cervix, posteriorly, instead of laterally, using the scissors and Emmet's knife.

Uterine Neuralgia.—To relieve ovarian and abdominal pain, due to chronic uterine inflammation, the following suppository will often be found to be very useful. It is made of one-quarter of a grain of the morphia sulphate, mixed with one grain of the alcoholic extract of belladonna. If the rectum has been previously emptied and well cleared, the absorption of the medicines will be speedy; it is an economical and effective mode of treatment. The recto-vaginal wall is so thin, that ovarian and vaginal inflammation and pain are often best treated by suppositories.

An ointment much used for ovarian and uterine inflammation and neuralgia, is the following:

Pulv. camphoræ, scr. j; ext. belladonnæ, dr. ij; unguenti hydrargyri and cerati simplicis, *aa* oz. ss. M. Ft. unguent. Take of this a small quantity and rub thoroughly over seat of pain.

Sometimes a plaster fulfills the indications more gratefully, and then the following may be used: Take a belladonna plaster, four by six inches (or of whatever size may be desired), and sprinkle upon it four grains of the morphia sulphate and one scruple of powdered camphor.

To act upon the sympathetic nervous system, the following is a routine Dispensary prescription, in diseases of women:

R. Potassii bromidi, gr. x; potassæ bicarbonatis, gr. xx; ext. gelsemini fluidi, gtt. ij; aquæ camphoræ, f. oz. j. M. Sig. Take the same, three times each day.

The formula is likewise useful in vesical irritation, complicating uterine troubles.

The Board of Health of the City of Chicago.—Since our visit, the Board, unwilling to remain longer in their narrow, inconvenient and unsuitable quarters, whence they had more than once been driven by torrents of rain breaking in through the roof and its crevices, have removed to commodious apartments in the Honore Building, corner Dearborn and Monroe Streets. We were informed that the past season has been remarkably destitute of any prominent feature of disease, except infantile diarrhœa, in which, however, the mortality has been very large. Probably, the mortality due to this cause, in children under five years of age, has been, at times, eighty per cent. of the entire weekly mortality.

The chief sanitary event this summer has been the laying down of a large amount of sewers, and enforcing the ordinance compelling connections to be made from each house with the main sewer. The districts have been made more salubrious, and the general adult health has been excellent. In the 7th, 8th and 9th wards, the contrast is very apparent.

Allusion was made to the indifference and laxity which accoucheurs, and especially midwives, exhibit in reporting births. It is apparent to all, how valuable these returns might become in many ways, even if not instantly available; as, for example, in a legal point of view, as a record to determine inheritance; also to determine the rate per cent. in different districts and cities, and occupations; also, in a moral point of view; in some of the New England States there are very few births, relatively, among married people.

The Board is now congratulating itself that it has no patient in the Small-Pox Hospital, and no new cases reported in the city. Lately, in vaccinating, the preference has been given to the use of human vaccine virus, one or two removes from the bovine: it is thought to operate more certainly and mildly; the bovine and vaccine lymph dries and hardens on the quill, and all physicians cannot, or at least do not, get it off.

DISINFECTANTS.—Take a shallow and broad saucer and partly cover it with chloride of lime, then pour upon it an half ounce of impure carbolic acid. To fumigate rooms after death, to the above add a little strong sulphuric acid. Carbolic acid is not now regarded with so much favor as heretofore; it is disagreeable and irritating to the lungs; it impregnates the food, and neighbors are annoyed by its odor.

